# Melatonin Attenuates Glucolipotoxicity-Induced Cardiac Oxidative Stress, Inflammation, Pyroptosis, and Fibrotic Remodeling in STZ/HFD-Treated ApoE^−^/^−^ Mice

**DOI:** 10.3390/antiox15070825

**Published:** 2026-06-30

**Authors:** Chia-Hui Lin, I-Ning Tsai, Ai-Ting Jou, Chau-Jong Wang, Ming-Chih Chou, Hui-Pei Huang, Chien-Ning Huang

**Affiliations:** 1 Institute of Medicine, Chung Shan Medical University, Taichung 402, Taiwan; ruby.chiahui@gmail.com (C.-H.L.); 880222sophia@gmail.com (A.-T.J.); mcchou@csmu.edu.tw (M.-C.C.); 2 Department of Health Diet and Industry Management, Chung Shan Medical University, Taichung 402, Taiwan; 0312elise@gmail.com (I.-N.T.); wcj@csmu.edu.tw (C.-J.W.); 3 Department of Medical Research, Chung Shan Medical University Hospital, Taichung 40201, Taiwan; 4 Division of Thoracic Surgery, Department of Surgery, Chung Shan Medical University Hospital, Taichung 40201, Taiwan; 5 School of Medicine, Chung Shan Medical University, Taichung 402, Taiwan; 6 Department of Family and Community Medicine, Chung Shan Medical University Hospital, Taichung 40201, Taiwan; 7 Department of Biochemistry, School of Medicine, Chung Shan Medical University, Taichung 40201, Taiwan; 8 Department of Clinical Laboratory, Chung Shan Medical University Hospital, Taichung 40201, Taiwan; 9 Division of Endocrinology and Metabolism, Department of Internal Medicine, Chung Shan Medical University Hospital, Taichung 40201, Taiwan

**Keywords:** melatonin, diabetic cardiomyopathy, oxidative stress, NLRP3 inflammasome, cardiac fibrosis, pyroptosis, glucolipotoxicity

## Abstract

Diabetic cardiomyopathy (DCM) under glucolipotoxic stress is sustained by a reactive oxygen species (ROS)-driven circuit in which oxidative DNA damage and nitrosative injury prime NLR family pyrin domain containing 3 (NLRP3) inflammasome assembly, triggering caspase-1 activation, gasdermin D (GSDMD) cleavage, and pyroptotic cardiomyocyte death that propagates apoptosis and fibrotic remodeling. Whether melatonin can disrupt this oxidative-pyroptotic axis when both hyperglycemia and dyslipidemia coexist, the metabolic context most refractory to current therapy has not been established. Apolipoprotein E-deficient (ApoE^−^/^−^) mice were subjected to streptozotocin-induced hyperglycemia and high-fat diet-induced dyslipidemia, then treated with oral melatonin (20 mg/kg/day) for 8 weeks. Despite unchanged fasting glycemia, melatonin attenuated cardiac oxidative stress, reducing 8-OHdG and inducible nitric oxide synthase while restoring Nrf2. Suppression of nuclear factor-κB and NLRP3 was accompanied by lowered interleukin-1β, caspase-1, and GSDMD, indicating disrupted inflammasome priming and pyroptotic execution. Downstream pathology was concurrently attenuated, with reduced TUNEL-positive cardiomyocytes, normalized Bax/Bcl-2 ratio, lower natriuretic peptide expression, diminished interstitial fibrosis, and improved electrocardiographic parameters. These findings position melatonin as a cardioprotective agent that operates despite persistent fasting hyperglycemia, acting through combined attenuation of lipid burden, cumulative glycemic stress, oxidative stress, and inflammatory signaling to arrest downstream apoptotic and fibrotic remodeling under glucolipotoxic conditions, providing a mechanistic rationale for adjunctive melatonin therapy in DCM.

## 1. Introduction

Diabetes mellitus (DM) is a global pandemic affecting more than 537 million individuals, with its prevalence continuing to rise [1]. Among the most devastating consequences of chronic DM is its impact on the cardiovascular system. Beyond macrovascular disease, DM directly impairs myocardial structure and function through a distinct pathological entity first described by Rubler et al. in 1972, termed diabetic cardiomyopathy (DCM) [2]. DCM is defined as ventricular dysfunction occurring independently of coronary artery disease, valvular abnormalities, or hypertension [3]. Its early stage is characterized by myocardial fibrosis, diastolic dysfunction, and pathological remodeling; without intervention, these changes progress to systolic dysfunction and overt heart failure [4]. The pathogenesis of DCM involves a complex interplay of metabolic dysregulation, oxidative stress, chronic sterile inflammation, cardiomyocyte apoptosis, and extracellular matrix remodeling [5].

Dyslipidemia frequently accompanies DM and substantially amplifies cardiovascular risk. Elevated total cholesterol, low-density lipoprotein (LDL) cholesterol, and triglycerides, together with reduced high-density lipoprotein (HDL) cholesterol, accelerate atherogenesis and exacerbate myocardial injury through enhanced oxidative and inflammatory signaling [4]. The apolipoprotein E-deficient (ApoE^−^/^−^) mouse is a well-validated model of hypercholesterolemia-driven atherosclerosis, spontaneously developing extensive vascular lesions even on a standard chow diet [6]. When challenged with streptozotocin (STZ) injection and high-fat diet (HFD) feeding, ApoE^−^/^−^ mice develop concurrent hyperglycemia and severe dyslipidemia, recapitulating the dual metabolic insult observed in type 2 diabetic patients with established cardiovascular risk [6]. This combined model therefore provides a pathophysiologically relevant platform for investigating diabetic cardiac disease.

Oxidative stress is widely recognized as a key contributor to the development and progression of DCM [7]. Under hyperglycemic and dyslipidemic conditions, excessive production of reactive oxygen species (ROS) leads to oxidative damage to cellular macromolecules, including DNA, lipids, and proteins, thereby impairing cardiac function [8]. Emerging evidence suggests that oxidative stress interacts with inflammatory signaling pathways, including NF-κB activation and NLRP3 inflammasome assembly, promoting chronic myocardial inflammation and structural remodeling [9,10]. Oxidative stress has further been associated with metabolic disturbances, cardiomyocyte apoptosis, and fibrotic remodeling, all of which contribute to the progression of cardiac dysfunction in diabetic conditions [11]. However, the integrated impact of oxidative stress on these pathological processes, particularly under combined glucolipotoxic stress, remains incompletely understood.

**Figure 1:**
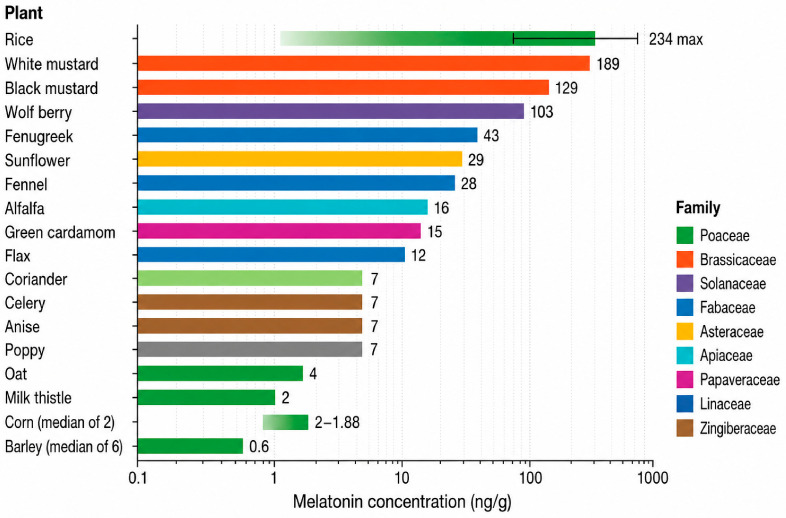
Conceptual overview of dietary melatonin sources and proposed cardioprotective mechanisms. The bar chart shows the reported concentrations of melatonin in various plant sources, including rice, white mustard, black mustard, wolf berry, fenugreek, sunflower, fennel, alfalfa, green cardamom, flax, coriander, celery, anise, poppy, oat, milk thistle, corn, and barley. Values are presented as melatonin concentration in ng/g. Different colors indicate plant families [13].

Melatonin (N-acetyl-5-methoxytryptamine) is an indoleamine predominantly produced by the pineal gland during the dark phase of the circadian cycle [12]. Beyond endogenous biosynthesis, melatonin is also widely distributed in edible plant sources, including cereals, seeds, nuts, fruits, and medicinal herbs, with reported tissue concentrations varying by several orders of magnitude across species. Dietary intake of melatonin-rich foods has been shown to elevate circulating melatonin and enhance systemic antioxidant capacity, supporting the relevance of melatonin as a nutraceutical with cardiometabolic implications (Figure 1) [13]. In addition to its role in circadian regulation, melatonin has been reported to function as a direct free radical scavenger and to enhance endogenous antioxidant defenses, including superoxide dismutase and glutathione peroxidase [14]. Melatonin has also been shown to exert anti-inflammatory effects, partly through suppression of NF-κB activation and inhibition of NLRP3 inflammasome signaling, thereby reducing IL-1β production and downstream inflammatory responses [15]. Previous studies have suggested that melatonin may attenuate cardiac fibrosis and apoptosis in diabetic conditions through multiple mechanisms [16]. However, these findings have largely been derived from models involving single metabolic insults.

Glucolipotoxicity refers to the synergistic cytotoxic effects of concurrent hyperglycemia and elevated circulating free fatty acids on cardiac tissue. This dual metabolic insult exceeds the injury attributable to either stimulus alone, amplifying ROS generation, promoting accumulation of toxic lipid intermediates such as diacylglycerols and ceramides, and activating mitochondrial damage pathways [17]. In cardiomyocytes, simultaneous exposure to elevated glucose and saturated fatty acids, particularly palmitic acid, induces intracellular ROS overproduction, glycogen deposition, lipid droplet accumulation, and cardiomyocyte apoptosis. These findings have been consistently reproduced in H9c2 rat cardiomyoblasts and recapitulate the histopathological features of clinical DCM [17]. These toxic lipid intermediates further activate inflammatory cascades and accelerate fibrotic remodeling, compounding the structural deterioration of the myocardium [18]. Critically, a substantial residual cardiovascular risk persists even when blood glucose is adequately controlled, underscoring the insufficiency of glucose-centric strategies in arresting cardiac disease progression [19]. This observation highlights the therapeutic importance of targeting oxidative and inflammatory pathways that operate downstream of metabolic dysregulation, independently of glycemic normalization.

The pleiotropic cardioprotective actions of melatonin, including ROS scavenging, suppression of NF-κB and NLRP3 inflammasome signaling, attenuation of apoptosis, and inhibition of fibrotic remodeling, operate principally at these downstream effector nodes [20,21]. Accordingly, melatonin may confer meaningful cardiac protection under glucolipotoxic stress even without normalizing blood glucose, a clinically relevant scenario in DCM patients receiving conventional antihyperglycemic therapy with incomplete cardiovascular benefit. However, the ability of melatonin to attenuate the compound myocardial injury induced by the concurrent presence of hyperglycemia and dyslipidemia has not been comprehensively investigated. The present study therefore examined the cardioprotective effects of melatonin in ApoE^−^/^−^ mice subjected to combined STZ-induced hyperglycemia and high-fat diet-induced dyslipidemia, focusing on glucolipotoxicity-driven oxidative stress, myocardial inflammation, cardiomyocyte apoptosis, and structural cardiac remodeling as primary outcome domains.

## 2. Materials and Methods

### 2.1. Animals and Experimental Design

Male C57BL/6J wild-type mice and C57BL/6-ApoE^−^/^−^ (ApoE^−^/^−^) mice, aged 4 weeks, were purchased from the National Laboratory Animal Center, National Applied Research Laboratories (Taipei, Taiwan). Animals were housed at the Chung Shan Medical University Laboratory Animal Center under controlled conditions (25 ± 1 °C, relative humidity 55 ± 5%, 12 h light/dark cycle) with ad libitum access to food and water. All animal experiments were approved by the Institutional Animal Care and Use Committee of Chung Shan Medical University (IACUC, CSMU; approval no. 112116). ApoE^−^/^−^ mice served as the model of cardiometabolic dysfunction, whereas age-matched C57BL/6J mice served as normal controls.

Hyperglycemia was induced by intraperitoneal injection of streptozotocin (STZ; 50 mg/kg/day) dissolved in 0.1 M sodium citrate buffer (pH 4.5) for 5 consecutive days [6]. One week after the final injection, animals with fasting blood glucose exceeding 200 mg/dL were classified as diabetic and enrolled in subsequent experiments [6]. Diabetic mice were then randomly assigned to experimental groups (*n* = 4 per group) and maintained on the assigned diet for 8 weeks, as follows:

1. Control, C: C57BL/6J mice fed a standard chow diet (PicoLab Rodent Diet 5010, LabDiet, St. Louis, MO, USA; 24.6% protein, 5.0% fat, 4.2% crude fiber, and 6.1% ash by weight).
2. Negative Control, NC: ApoE^−^/^−^ mice maintained on a standard chow diet.
3. STZ: ApoE^−^/^−^ mice administered STZ and fed a standard chow diet.
4. STZ + HFD: ApoE^−^/^−^ mice administered STZ and fed a high-fat atherogenic diet (17% lard, 1.2% cholesterol, and 0.2% sodium cholate hydrate).
5. STZ + HFD + MT: ApoE^−^/^−^ mice administered STZ and HFD, and treated with melatonin (20 mg/kg/day).

This combined hyperglycemic and dyslipidemic regimen reproduces the glucolipotoxic vascular phenotype described for STZ-treated ApoE^−^/^−^ mice [22]. Melatonin was dissolved in sterile water and administered by oral gavage throughout the 8-week treatment period, consistent with a previously reported cardioprotective dosing protocol in diabetic cardiomyopathy. Body weight was recorded weekly, and fasting blood glucose was measured every 2 weeks using a glucometer.

### 2.2. Blood Biochemical Analysis

At sacrifice, blood was collected by cardiac puncture under CO_2_ anesthesia. Samples were centrifuged at 3000× *g* for 5 min, and serum was stored at −80 °C until analysis. Serum concentrations of total cholesterol (CHO), triglycerides (TGs), high-density lipoprotein cholesterol (HDL-C), and low-density lipoprotein cholesterol (LDL-C) were quantified using commercial enzymatic colorimetric kits according to the manufacturer’s protocols. Glycated hemoglobin (HbA1c) was determined using a Mouse Hemoglobin A1c (HbA1c) Assay Kit (Cat. No. 80310; Crystal Chem, Elk Grove Village, IL, USA).

### 2.3. Electrocardiography

Cardiac electrophysiological function was assessed 24 h prior to sacrifice using a small animal surgical monitoring platform (Rodent Surgical Monitor+, RSM+). Lead II electrocardiography (ECG) was recorded by measuring the potential difference between the left hindlimb and right forelimb [23]. PQRST waveforms were analyzed for heart rate (bpm), RR interval, PR interval, QRS interval, P-wave amplitude, and R-wave amplitude using ImageJ version 1.54g. (NIH, Bethesda, MD, USA). Statistical analysis was performed using SigmaPlot 12.0.

### 2.4. Heart Tissue Harvest and Gross Morphology

Following cardiac blood collection, hearts were excised, rinsed with phosphate-buffered saline (PBS), blotted dry, and photographed for gross morphological assessment. Heart weight was recorded, and the heart-to-body-weight ratio was calculated for each animal. Cardiac tissue designated for histological analysis was fixed in 10% neutral buffered formalin and embedded in paraffin.

### 2.5. Histological Examination

Paraffin-embedded cardiac sections (5 μm) were deparaffinized in xylene and rehydrated through a graded ethanol series (100%, 95%, 80%, 70%) to distilled water. Three staining protocols were applied. Hematoxylin and eosin (H&E) staining was used to evaluate myofibrillar architecture, cardiomyocyte morphology, and leukocyte infiltration. Sections were counterstained with hematoxylin and eosin according to standard protocols [24]. Periodic acid–Schiff (PAS) staining was applied to assess glycogen and neutral polysaccharide accumulation within cardiomyocytes. Abnormal glycogen and lipid droplet deposition is a recognized histopathological feature of glucolipotoxicity myocardial injury [25]. PAS-positive signal (magenta granules) was quantified as percentage staining area using ImageJ. Masson’s trichrome staining was performed to visualize and quantify interstitial collagen deposition and fibrotic remodeling. Blue-stained collagen fibers were expressed as percentage of total tissue area using ImageJ [26]. All sections were digitized using a TissueGnostics tissue cytometer (TissueGnostics, Vienna, Austria).

### 2.6. Immunohistochemical Staining

Paraffin-embedded cardiac sections (5 μm) were deparaffinized and rehydrated as described above. Antigen retrieval was performed by heating in 0.01 M citrate buffer (pH 6.0) at approximately 100 °C for 10 min, followed by cooling to room temperature. Endogenous peroxidase activity was quenched with 3% hydrogen peroxide in methanol. Sections were blocked with 1% fetal bovine serum (FBS) in TBST for 1 h at room temperature. Primary antibodies diluted in 0.5% FBS/TBST were incubated at 37 °C for 2 h. The following targets were assessed: 8-OHdG, a marker of oxidative DNA damage [27]; inducible nitric oxide synthase (iNOS), a marker of nitrosative stress [28]; atrial natriuretic peptide (ANP) and brain natriuretic peptide (BNP), complementary indices of atrial and ventricular myocardial wall stress [29]; tumor necrosis factor-α (TNF-α); NF-κB; NLRP3; and IL-1β [9]. After TBST washing, diaminobenzidine (DAB) chromogen substrate (DAB:H_2_O_2_ = 1:1) was applied for 10 min at room temperature in the dark. Hematoxylin counterstaining was applied for nuclear visualization. Slides were dehydrated, cleared in xylene, and cover slipped. Immunoreactivity was quantified by integrated optical density (IOD) using ImageJ and expressed as percentage of control.

### 2.7. Immunofluorescence Staining

Paraffin-embedded cardiac sections (5 μm) were deparaffinized, rehydrated, and subjected to heat-induced antigen retrieval in 0.01 M citrate buffer (pH 6.0) as described above. Sections were permeabilized with 0.1% Triton X-100 in PBS for 10 min at room temperature and blocked with 5% fetal bovine serum (FBS) in TBST for 1 h at room temperature. Primary antibodies diluted in 1% FBS/TBST were applied and incubated at 4 °C overnight in a humidified chamber. The following targets were assessed: nuclear factor erythroid 2-related factor 2 (Nrf2), the master transcriptional regulator of the endogenous antioxidant response [30]; cleaved caspase-1 and gasdermin D (GSDMD), executioners of NLRP3 inflammasome-mediated pyroptosis [9]; and the pro-apoptotic protein Bax together with the anti-apoptotic protein Bcl-2, complementary indices of the mitochondrial apoptotic set-point [31]. For dual labeling, primary antibodies raised in different host species were co-incubated. After three TBST washes, sections were incubated with Alexa Fluor 488- and Alexa Fluor 594-conjugated species-matched secondary antibodies in 1% FBS/TBST for 1 h at room temperature in the dark. Nuclei were counterstained with 4′,6-diamidino-2-phenylindole (DAPI) for 5 min at room temperature. Sections were washed, mounted with anti-fade mounting medium, and imaged using a ZEISS Axio Imager upright fluorescence microscope (Carl Zeiss, Oberkochen, Germany). Antibody sources, catalog numbers, and working dilutions are summarized in Supplementary Table S1. Fluorescence signal was quantified by integrated optical density (IOD) using ImageJ and expressed as percentage of control. For each section, five randomly selected non-overlapping fields were analyzed and averaged per animal.

### 2.8. TUNEL Assay

Cardiomyocyte apoptosis was assessed by terminal deoxynucleotidyl transferase dUTP nick-end labeling (TUNEL) using a commercially available fluorescence detection kit. TUNEL is a validated method for the in situ detection of DNA strand breaks in apoptotic cardiomyocytes within fixed cardiac tissue sections [32]. Following deparaffinization and rehydration, sections were incubated with proteinase K working solution (1×) at 37 °C for 20 min, washed with TBST, and equilibrated with TdT Equilibration Buffer at 37 °C for 30 min. The labeling working solution was then applied at 37 °C for 60 min in the dark. After TBST washes, the DAPI working solution was applied for 5 min at room temperature in the dark for nuclear counterstaining. Slides were washed, coverslipped, and imaged using a ZEISS Axio Imager upright fluorescence microscope.

### 2.9. Statistical Analysis

All data are expressed as mean ± SEM. Statistical analyses were performed using SigmaPlot 12.0 (Systat Software Inc., Chicago, IL, USA). Between-group comparisons were made using one-way analysis of variance (ANOVA) followed by Duncan’s multiple range test. Student’s *t*-test was applied for selected pairwise comparisons. Statistical significance was defined as *p* < 0.05. Sample sizes per group are indicated in each figure or table legend.

## 3. Results

### 3.1. Melatonin Improved Blood Biochemical Parameters in STZ- and HFD-Induced Diabetic ApoE^−^/^−^ Mice

To evaluate the systemic metabolic burden induced by combined STZ injection and high-fat diet, serum biochemical parameters were analyzed after 8 weeks of treatment (Table 1). Total cholesterol was markedly elevated in all ApoE^−^/^−^-derived groups (822.6 ± 100.25 to 1518.8 ± 102.26 mg/dL) compared with C57BL/6 controls (94.6 ± 17.32 mg/dL), consistent with the hypercholesterolemic phenotype of ApoE deficiency.

Compared with the ApoE^−^/^−^ group, the STZ + HFD group exhibited significant elevations in triglyceride (136.2 ± 16.60 vs. 86.8 ± 14.60 mg/dL, *p* < 0.01), LDL cholesterol (244.6 ± 43.46 vs. 111.8 ± 9.36 mg/dL, *p* < 0.001), and HbA1c (6.8 ± 0.70% vs. 3.9 ± 0.08%, *p* < 0.001), confirming a glucolipotoxic metabolic phenotype. Melatonin treatment significantly reduced triglyceride to 89.6 ± 12.81 mg/dL (*p* < 0.01 vs. STZ + HFD), LDL cholesterol to 157.6 ± 42.12 mg/dL (*p* < 0.01 vs. STZ + HFD), and HbA1c to 5.1 ± 0.76% (*p* < 0.01 vs. STZ + HFD). HDL cholesterol was elevated in all ApoE^−^/^−^-derived groups consistent with the genotype, with no significant difference among diabetic and melatonin-treated groups. These results indicate that melatonin ameliorates dyslipidemia and cumulative glycemic burden in STZ + HFD-induced diabetic ApoE^−^/^−^ mice.

### 3.2. Melatonin Prevents Diabetes-Associated Cardiac Hypertrophy

**Figure 2:**
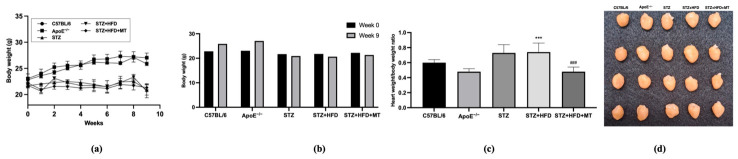
Effects of melatonin on body weight, heart weight, and gross cardiac morphology in STZ-diabetic ApoE^−^/^−^ mice. (**a**) Changes in body weight during the experimental period. (**b**) Comparison of initial and final body weights. (**c**) Heart weight/body weight ratio. (**d**) Representative gross images of hearts from each experimental group. C57BL/6 mice fed a normal diet served as the control group, while ApoE^−^/^−^ mice fed a normal diet served as the negative control group. Diabetes was induced in ApoE^−^/^−^ mice by intraperitoneal injection of streptozotocin (STZ). The STZ + HFD group received STZ injection combined with a high-fat diet, whereas the STZ + HFD + MT group received STZ injection, high-fat diet, and oral administration of melatonin at 20 mg/kg for 8 weeks. Body weight was monitored throughout the experimental period, and initial and final body weights were compared to assess weight changes during the intervention. Heart weight and gross cardiac morphology were examined at the end of the experiment. Data are expressed as mean ± SD. *** *p* < 0.001 compared with the ApoE^−^/^−^ group; ^###^ *p* < 0.001 compared with the STZ + HFD group.

To determine whether melatonin affected diabetes-associated cardiac remodeling, body weight, heart weight, and gross cardiac morphology were examined (Figure 2). Body weight increased progressively in the C57BL/6 and ApoE^−^/^−^ groups, reaching approximately 27 g by the end of the experimental period. In contrast, the STZ-treated groups maintained lower body weights of approximately 21–22 g throughout the study. Comparison of the initial and final body weights showed no significant increase in the STZ, STZ + HFD, or STZ + HFD + MT groups, indicating that STZ-induced diabetes prevented normal body weight gain despite HFD feeding (Figure 2a,b). Heart weight was significantly increased in the STZ and STZ + HFD groups compared with the ApoE^−^/^−^ group, with no significant difference between the STZ and STZ + HFD groups. Because body weight was reduced in the diabetic groups, we further calculated the heart weight/body weight ratio as an additional index of relative cardiac enlargement. The heart weight/body weight ratio was increased in the STZ and STZ + HFD groups, suggesting diabetes-associated adverse cardiac remodeling rather than physiological growth. Melatonin treatment reduced both heart weight and the heart weight/body weight ratio to levels comparable to the ApoE^−^/^−^ group. Representative gross heart images are shown for morphological reference only and were not used as the primary basis for the quantitative conclusion (Figure 2c,d).

### 3.3. Melatonin Reduced Hyperinsulinemia Without Lowering Fasting Glucose

**Figure 3:**
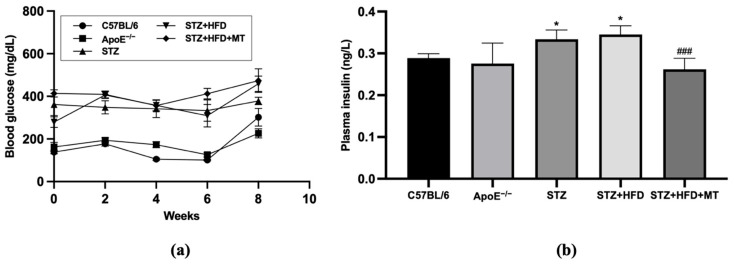
Effects of melatonin on blood glucose and plasma insulin levels in STZ-diabetic ApoE^−^/^−^ mice. (**a**) Blood glucose levels were measured at 0, 2, 4, 6, and 8 weeks in C57BL/6, ApoE^−^/^−^, STZ, STZ + HFD, and STZ + HFD + MT mice. (**b**) Plasma insulin levels were measured at the end of the experimental period in the same groups. C57BL/6 mice fed a normal diet served as the control group. ApoE^−^/^−^ mice fed a normal diet served as the negative control group. Diabetes was induced in ApoE^−^/^−^ mice by intraperitoneal injection of streptozotocin (STZ). The STZ + HFD group received STZ injection combined with a high-fat diet, whereas the STZ + HFD + MT group received STZ injection, high-fat diet, and oral administration of melatonin at 20 mg/kg for 8 weeks. Blood glucose levels were monitored throughout the experimental period, and plasma insulin levels were measured at the end of the experiment. Blood glucose levels were analyzed using two-way repeated-measures ANOVA followed by post hoc comparisons; no statistically significant differences were detected among groups at the measured time points. Data are expressed as mean ± SD. * *p* < 0.05 compared with the ApoE^−^/^−^ group; ^###^ *p* < 0.001 compared with the STZ + HFD group.

To assess whether the protective effects of melatonin involved changes in glucose homeostasis, fasting blood glucose was monitored throughout the experimental period and plasma insulin was measured at week 8 (Figure 3). Fasting blood glucose remained markedly elevated in all STZ-injected groups (approximately 350–470 mg/dL) compared with C57BL/6 and ApoE^−^/^−^ controls (approximately 150–200 mg/dL) throughout the study, and melatonin did not decrease fasting glucose in the STZ + HFD + MT group.

Plasma insulin was significantly increased in the STZ (0.33 ± 0.02 ng/L, *p* < 0.05) and STZ + HFD (0.345 ± 0.02 ng/L, *p* < 0.05) groups compared with the ApoE^−^/^−^ group (0.27 ± 0.05 ng/L), indicating compensatory hyperinsulinemia under glucolipotoxic stress. Melatonin treatment significantly reduced plasma insulin to 0.26 ± 0.02 ng/L (*p* < 0.001 vs. STZ + HFD). These findings indicate that melatonin attenuates hyperinsulinemia despite persistent fasting hyperglycemia, suggesting improved peripheral insulin handling rather than direct correction of basal glycemia.

### 3.4. Melatonin Preserves Myocardial Architecture and Limits Fibrotic Remodeling

**Figure 4:**
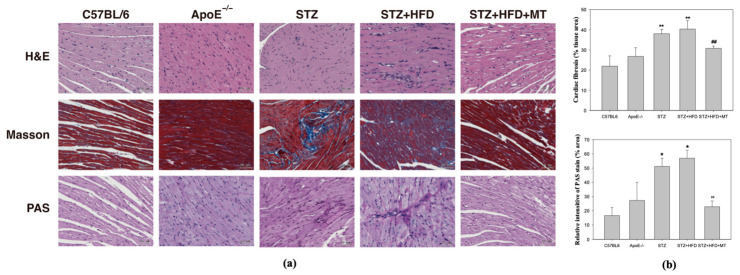
Melatonin attenuated histopathological changes, cardiac fibrosis, and PAS-positive staining in the hearts of STZ-diabetic ApoE^−^/^−^ mice. (**a**) Representative cardiac sections were stained with hematoxylin and eosin (H&E), Masson’s trichrome, and periodic acid–Schiff (PAS) staining. Images were captured at the indicated magnification, and scale bars represent 50 μm. H&E staining was used to evaluate cardiac morphology and myocardial structural alterations. Masson’s trichrome staining was used to assess cardiac fibrosis, and PAS staining was used to evaluate PAS-positive myocardial staining. (**b**) Quantitative analysis of cardiac fibrosis, and PAS staining. The STZ and STZ + HFD groups showed aggravated myocardial pathological changes, increased collagen deposition, and enhanced PAS-positive staining compared with the ApoE^−^/^−^ group. Melatonin treatment reduced cardiac fibrosis and PAS-positive staining in the STZ + HFD + MT group. Quantitative results are shown as cardiac fibrosis percentage and relative intensity of PAS staining. Data are expressed as mean ± SD. * *p* < 0.05 and ** *p* < 0.01 compared with the ApoE^−^/^−^ group; ^##^ *p* < 0.01 compared with the STZ + HFD group.

To evaluate the structural impact of glucolipotoxicity and melatonin on the myocardium, hematoxylin and eosin (H&E), Masson’s trichrome, and periodic acid–Schiff (PAS) staining were performed (Figure 4). H&E staining revealed organized myocardial architecture in C57BL/6 and ApoE^−^/^−^ groups, whereas STZ and STZ + HFD groups exhibited myofibrillar disorganization, inflammatory cell infiltration, and disrupted alignment. Melatonin treatment restored myocardial morphology in the STZ + HFD + MT group.

Masson’s trichrome staining showed increased collagen deposition in the STZ (38.0 ± 2.5%, *p* < 0.01) and STZ + HFD (40.3 ± 3.1%, *p* < 0.01) groups compared with the ApoE^−^/^−^ group (26.7 ± 4.0%). Melatonin treatment significantly reduced the cardiac fibrotic area to 30.8 ± 1.9% (*p* < 0.01 vs. STZ + HFD).

PAS staining was used to evaluate myocardial substrate accumulation. The PAS-positive area was markedly increased in the STZ (51.3 ± 5.6%, *p* < 0.05) and STZ + HFD (57.2 ± 6.3%, *p* < 0.05) groups compared with the ApoE^−^/^−^ group (27.0 ± 7.5%), reflecting glycogen and polysaccharide accumulation under diabetic conditions. Melatonin treatment significantly reduced the PAS-positive area to 22.9 ± 3.1% (*p* < 0.01 vs. STZ + HFD). These findings indicate that melatonin attenuates glucolipotoxicity-induced histopathological injury, fibrosis, and abnormal substrate accumulation in diabetic hearts.

### 3.5. Melatonin Restores Cardiac Electrical Conduction Under Glucolipotoxic Stress

**Figure 5:**
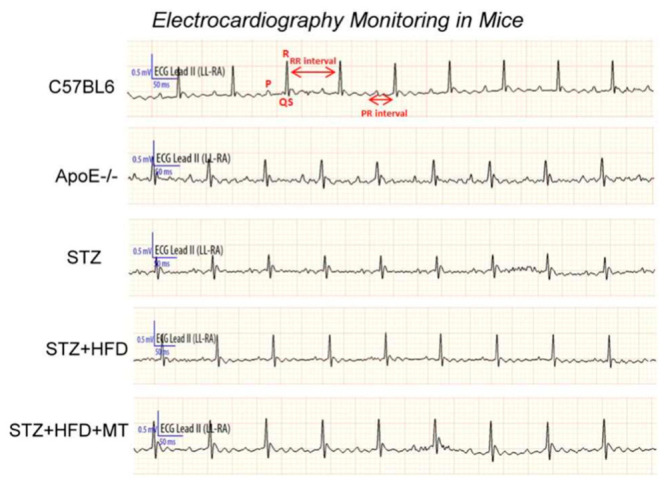
Representative electrocardiographic tracings in STZ-diabetic ApoE^−^/^−^ mice. Representative lead II electrocardiographic tracings were recorded from C57BL/6, ApoE^−^/^−^, STZ, STZ + HFD, and STZ + HFD + MT groups. C57BL/6 mice were fed a normal diet as the control group, whereas ApoE^−^/^−^ mice were fed a normal diet as the negative control group. STZ-diabetic ApoE^−^/^−^ mice were induced by intraperitoneal injection of streptozotocin. The STZ + HFD group received STZ injection combined with a high-fat diet, and the STZ + HFD + MT group received STZ injection, high-fat diet, and oral administration of melatonin at 20 mg/kg for 8 weeks. ECG tracings were used to evaluate heart rate, RR interval, PR interval, QRS interval, P amplitude, and R amplitude.

To investigate whether melatonin preserves cardiac electrical function under glucolipotoxic stress, lead II electrocardiographic recordings were performed at week 8 (Figure 5) and ECG parameters were quantified (Table 2). Compared with the ApoE^−^/^−^ group, the STZ + HFD group exhibited a significant decrease in heart rate and P-wave amplitude, accompanied by a prolonged RR interval, indicating impaired cardiac rhythm and atrial electrical activity. Melatonin treatment significantly restored heart rate and increased P-wave and R-wave amplitudes compared with the STZ + HFD group, suggesting improved atrial and ventricular electrical activity. Although the QRS interval only showed a recovery trend, these findings indicate that melatonin partially ameliorated STZ/HFD-induced ECG abnormalities in diabetic ApoE^−^/^−^ mice.

### 3.6. Melatonin Improves Cardiac Natriuretic Peptide Overexpression

**Figure 6:**
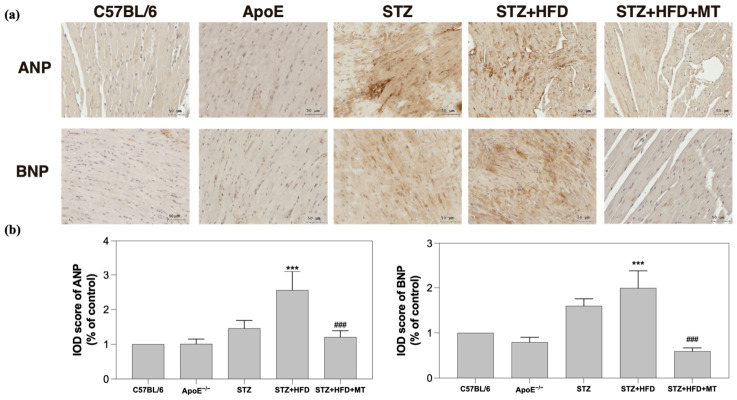
Melatonin reduced cardiac ANP and BNP expression in STZ-diabetic ApoE^−^/^−^ mice. (**a**) Representative immunohistochemical images of atrial natriuretic peptide (ANP; upper panels) and brain natriuretic peptide (BNP; lower panels) in left ventricular tissues from C57BL/6 (C), ApoE^−^/^−^, STZ, STZ + HFD, and STZ + HFD + MT groups. Scale bar = 50 µm. (**b**) Quantitative integrated optical density (IOD) is expressed as percentage of control. STZ + HFD mice showed markedly elevated ANP and BNP immunoreactivity compared with ApoE^−^/^−^ mice, consistent with enhanced cardiomyocyte stress and hypertrophic response. Melatonin treatment attenuated these elevations. Data are presented as mean ± SD (*n* = 4). *** *p* < 0.001 versus the ApoE^−^/^−^ group; ^###^ *p* < 0.001 versus the STZ + HFD group.

To determine whether glucolipotoxicity increased myocardial stress, ANP and BNP expression in cardiac tissues was examined by immunohistochemistry (Figure 6). Both natriuretic peptides are established biomarkers of cardiomyocyte stretch, pressure overload, and pathological hypertrophy, with elevated myocardial expression reflecting adverse remodeling under metabolic stress [33]. ANP and BNP immunoreactivity remained faint in the C57BL/6 and ApoE^−^/^−^ groups. In contrast, the STZ and STZ + HFD groups displayed pronounced cytoplasmic staining within cardiomyocytes, with peak intensity in the STZ + HFD group. Melatonin treatment markedly diminished this staining. Quantitative IOD analysis was consistent with the staining pattern. ANP expression increased approximately 1.5-fold in STZ and 2.6-fold in STZ + HFD (*p* < 0.001) compared with the ApoE^−^/^−^ group. BNP expression increased approximately 2.1-fold in STZ and 2.6-fold in STZ + HFD (*p* < 0.001). Melatonin reduced ANP and BNP expression by approximately 53% and 70%, respectively, relative to the STZ + HFD group (both *p* < 0.001). These findings indicate that melatonin alleviates the natriuretic peptide signature of cardiac stress and hypertrophic remodeling under glucolipotoxic conditions.

### 3.7. Melatonin Attenuated Oxidative DNA Damage and Enhanced the Nrf2/HO-1 Antioxidant Pathway in Diabetic Myocardium Under Glucolipotoxic Stress

**Figure 7:**
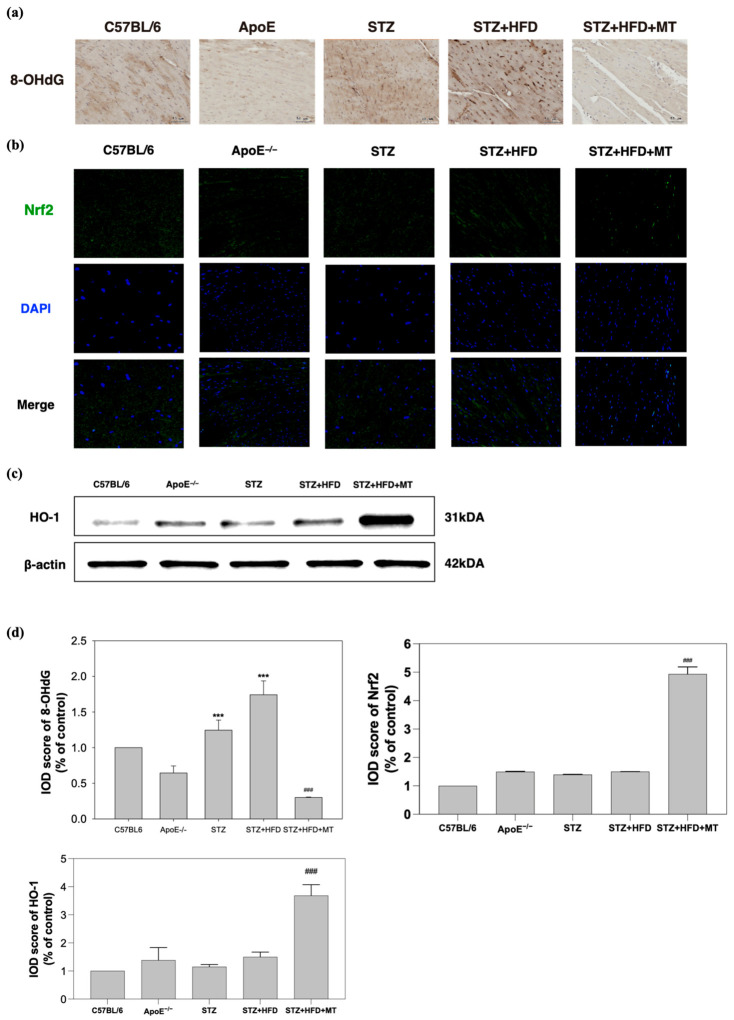
Melatonin attenuated oxidative DNA damage and activated the Nrf2/HO-1 antioxidant pathway in the hearts of STZ-diabetic ApoE^−^/^−^ mice. (**a**) Representative immunohistochemical staining images of 8-OHdG in cardiac tissues. Scale bar = 50 µm. (**b**) Representative immunofluorescence staining images of Nrf2 (green), DAPI nuclear counterstaining (blue), and merged images. (**c**) Representative Western blot images of HO-1 expression. (**d**) Quantitative analysis of 8-OHdG, Nrf2 immunoreactivity, and HO-1 protein expression. Melatonin strongly enhanced Nrf2 immunoreactivity and increased HO-1 expression in the STZ + HFD + MT group, suggesting activation of the Nrf2/HO-1 antioxidant defense pathway. Data are expressed as mean ± SD. *** *p* < 0.001 compared with the ApoE^−^/^−^ group; ^###^ *p* < 0.001 compared with the STZ + HFD group.

To evaluate oxidative DNA damage in diabetic hearts, cardiac 8-OHdG expression was examined by immunohistochemical staining (Figure 7a,d). The 8-OHdG immunoreactivity was low in the C57BL/6 and ApoE^−^/^−^ groups, whereas the STZ and STZ + HFD groups showed pronounced nuclear staining, indicating increased oxidative DNA damage under diabetic and glucolipotoxic conditions. Quantitative IOD analysis showed that 8-OHdG expression was significantly increased in the STZ and STZ + HFD groups compared with the ApoE^−^/^−^ group. Melatonin treatment markedly reduced 8-OHdG expression in the STZ + HFD + MT group, indicating attenuation of oxidative DNA damage in the diabetic myocardium.

To further assess the antioxidant response, Nrf2 expression was examined by immunofluorescence staining, and HO-1 expression, a canonical downstream target of Nrf2, was evaluated by Western blotting (Figure 7b–d). Nrf2 immunoreactivity was not markedly increased in the STZ and STZ + HFD groups despite the presence of oxidative DNA damage, suggesting an insufficient endogenous antioxidant response under glucolipotoxic stress. In contrast, melatonin treatment markedly enhanced Nrf2 immunoreactivity in the STZ + HFD + MT group. Consistently, HO-1 protein expression was also significantly increased after melatonin treatment. These findings suggest that melatonin attenuates glucolipotoxicity-induced oxidative DNA damage and reinforces the Nrf2/HO-1 antioxidant defense pathway in diabetic myocardium. However, because EMSA was not performed, the present data should be interpreted as supportive evidence of enhanced Nrf2/HO-1 antioxidant signaling rather than direct proof of Nrf2 DNA-binding activity.

### 3.8. Melatonin Suppressed Inflammatory and Inflammasome-Associated Markers Under Glucolipotoxic Stress

**Figure 8:**
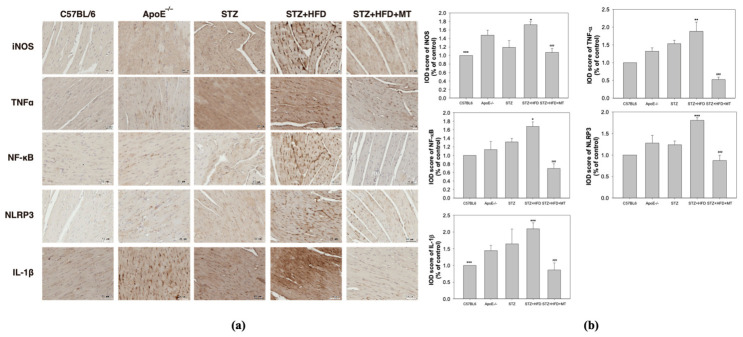
Melatonin suppressed inflammatory and inflammasome-related markers in cardiac tissues of STZ-diabetic ApoE^−^/^−^ mice. (**a**) Representative immunohistochemical staining images of inducible nitric oxide synthase (iNOS), tumor necrosis factor-α (TNF-α), nuclear factor-κB (NF-κB), NLR family pyrin domain containing 3 (NLRP3), and interleukin-1β (IL-1β) in cardiac tissues are shown. STZ and STZ + HFD groups exhibited increased expression of inflammatory and inflammasome-related markers compared with the ApoE^−^/^−^ group. Melatonin treatment reduced iNOS, TNF-α, NF-κB, NLRP3, and IL-1β expression in the STZ + HFD + MT group. Scale bar = 50 µm. (**b**) Quantitative IOD analysis was performed and expressed as percentage of control. Data are expressed as mean ± SD. * *p* < 0.05, ** *p* < 0.01, and *** *p* < 0.001 compared with the ApoE^−^/^−^ group; ^###^ *p* < 0.001 compared with the STZ + HFD group; *n* = 4 per group.

To assess inflammatory and inflammasome-associated signaling in diabetic hearts, immunohistochemical staining for inducible nitric oxide synthase (iNOS), tumor necrosis factor-α (TNF-α), nuclear factor-κB (NF-κB), NLR family pyrin domain containing 3 (NLRP3), and interleukin-1β (IL-1β) were quantified in cardiac tissues (Figure 8). Compared with the ApoE^−^/^−^ group, the STZ + HFD group showed significantly increased expression of iNOS (1.7-fold, *p* < 0.05), TNF-α (1.9-fold, *p* < 0.01), NF-κB (1.65-fold, *p* < 0.05), NLRP3 (1.8-fold, *p* < 0.001), and IL-1β (2.1-fold, *p* < 0.001), indicating coordinated activation of upstream inflammatory priming and downstream inflammasome-mediated cytokine maturation. The STZ-only group showed intermediate elevations of these markers, consistent with a synergistic effect of combined hyperglycemia and dyslipidemia on myocardial inflammatory signaling.

Melatonin treatment significantly reduced expression of all five markers in the STZ + HFD + MT group: iNOS to 1.05-fold (*p* < 0.001 vs. STZ + HFD), TNF-α to 0.5-fold (*p* < 0.001 vs. STZ + HFD), NF-κB to 0.7-fold (*p* < 0.001 vs. STZ + HFD), NLRP3 to 0.85-fold (*p* < 0.001 vs. STZ + HFD), and IL-1β to 0.85-fold (*p* < 0.001 vs. STZ + HFD). These findings indicate that melatonin coordinately suppresses NF-κB-mediated transcriptional priming and NLRP3 inflammasome assembly, thereby attenuating IL-1β maturation and the pro-inflammatory cytokine response in glucolipotoxic myocardium.

### 3.9. Melatonin Suppressed Pyroptosis-Associated Signaling Under Glucolipotoxic Stress

**Figure 9:**
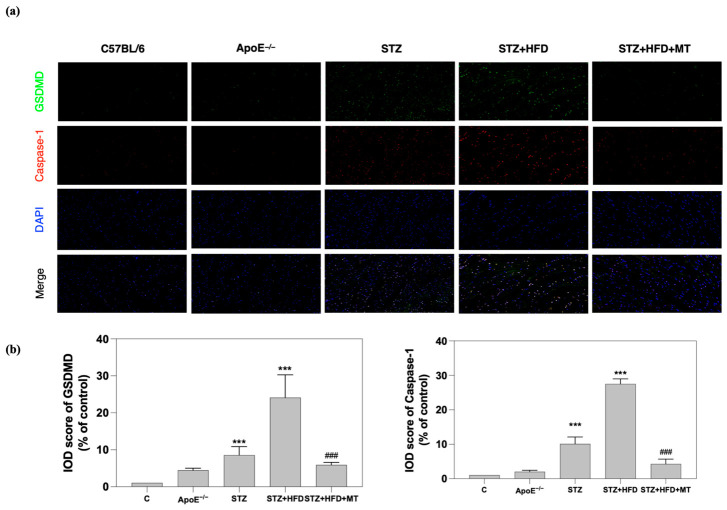
Effects of melatonin on pyroptosis-related markers in cardiac tissues of STZ-diabetic ApoE^−^/^−^ mice. (**a**) Representative immunofluorescence staining of gasdermin D (GSDMD, green) and cleaved caspase-1 (red) in cardiac tissues from C57BL/6, ApoE^−^/^−^, STZ, STZ + HFD, and STZ + HFD + MT groups. Nuclei were counterstained with DAPI (blue), and merged images are shown. Scale bar = 100 μm. The STZ and STZ + HFD groups exhibited increased GSDMD and caspase-1 fluorescence intensity compared with the ApoE^−^/^−^ group. Melatonin treatment reduced both signals in the STZ + HFD + MT group. (**b**) Quantitative analysis of integrated optical density (IOD) is expressed as percentage of control. Data are expressed as mean ± SD. *** *p* < 0.001 compared with the ApoE^−^/^−^ group; ^###^ *p* < 0.001 compared with the STZ + HFD group; *n* = 4 per group.

To determine whether NLRP3 inflammasome activation was accompanied by pyroptotic execution in the glucolipotoxic myocardium, the expression of cleaved caspase-1 and GSDMD was evaluated in cardiac tissues (Figure 9). Caspase-1 and GSDMD signals were low in the C57BL/6 and ApoE^−^/^−^ groups. The STZ and STZ + HFD groups exhibited prominent perinuclear and membrane-associated immunofluorescence, indicating activation of pyroptotic signaling under glucolipotoxic conditions. Caspase-1 fluorescence intensity was elevated in the STZ (4.97-fold, *p* < 0.001) and STZ + HFD (13.55-fold, *p* < 0.001) groups compared with the ApoE^−^/^−^ group. GSDMD signal showed a parallel increase in the STZ (1.92-fold, *p* < 0.001) and STZ + HFD (5.43-fold, *p* < 0.001) groups. Melatonin treatment reduced caspase-1 to 2.10-fold (*p* < 0.001 vs. STZ + HFD) and GSDMD to 1.33-fold (*p* < 0.001 vs. STZ + HFD) of the ApoE^−^/^−^ level. These results indicate that melatonin suppresses caspase-1/GSDMD-mediated pyroptotic signaling downstream of NLRP3 inflammasome activation in the glucolipotoxic myocardium.

### 3.10. Melatonin Protects Cardiomyocytes from Glucolipotoxic Apoptosis

**Figure 10:**
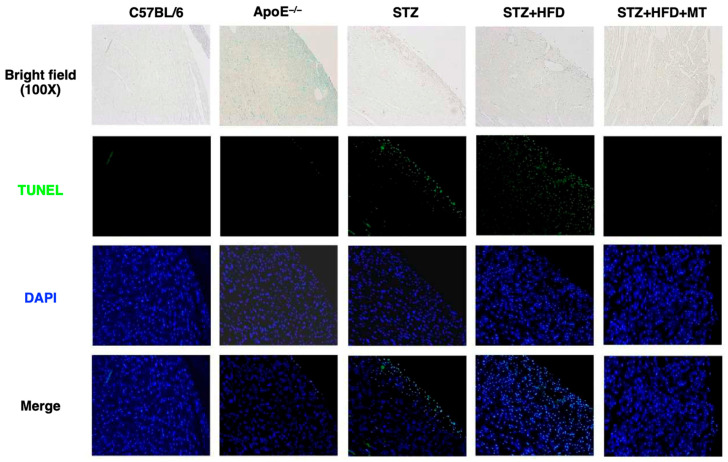
Melatonin reduced apoptosis in cardiac tissues of STZ-diabetic ApoE^−^/^−^ mice. Paraffin-embedded cardiac sections from C57BL/6, ApoE^−^/^−^, STZ, STZ + HFD, and STZ + HFD + MT groups were stained using a TUNEL assay. Representative bright-field, TUNEL, DAPI, and merged images are shown. Green indicates TUNEL-positive apoptotic cells, and blue indicates DAPI-stained nuclei. TUNEL-positive apoptotic cells were increased in the STZ and STZ + HFD groups compared with the ApoE^−^/^−^ group, whereas melatonin treatment reduced TUNEL-positive signals in the STZ + HFD + MT group.

To evaluate cardiomyocyte apoptosis, TUNEL staining was performed in cardiac sections from each experimental group (Figure 10). Few TUNEL-positive cells were observed in the C57BL/6 and ApoE^−^/^−^ groups. In contrast, TUNEL-positive apoptotic cells were increased in the STZ and STZ + HFD groups, with more pronounced signals observed under combined glucolipotoxic conditions. Melatonin treatment reduced TUNEL-positive signals in the STZ + HFD + MT group compared with the STZ + HFD group. These results suggest that melatonin attenuated diabetes-associated apoptotic injury in cardiac tissues.

**Figure 11:**
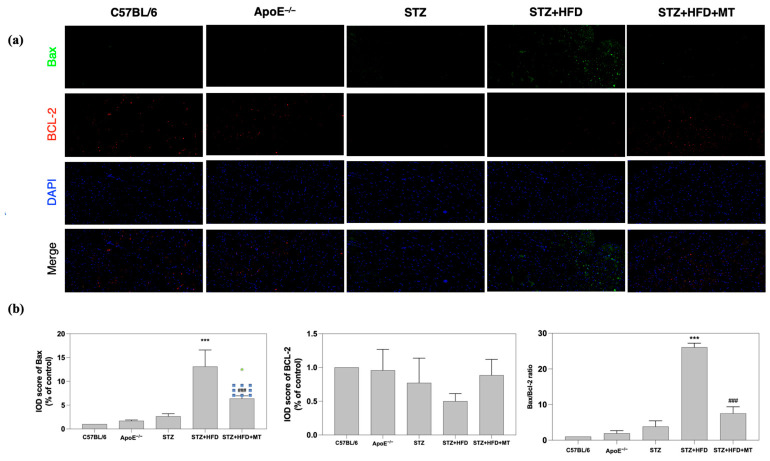
Effects of melatonin on apoptosis-related Bax and Bcl-2 expression in cardiac tissues of STZ-diabetic ApoE^−^/^−^ mice. (**a**) Representative immunofluorescence staining images of the pro-apoptotic protein Bax (green), the anti-apoptotic protein Bcl-2 (red), and DAPI nuclear counterstaining (blue), together with merged images, are shown for the C57BL/6, ApoE^−^/^−^, STZ, STZ + HFD, and STZ + HFD + MT groups. STZ and STZ + HFD groups exhibited increased Bax expression and decreased Bcl-2 expression compared with the ApoE^−^/^−^ group, whereas melatonin treatment attenuated Bax upregulation and restored Bcl-2 expression in the STZ + HFD + MT group. (**b**) Quantitative analysis of Bax expression, Bcl-2 expression, and the Bax/Bcl-2 ratio was performed and expressed as percentage of control. Bax is shown in green, BCL-2 is shown in red, and DAPI nuclear staining is shown in blue. Data are expressed as mean ± SD. *** *p* < 0.001 compared with the ApoE^−^/^−^ group; ^###^ *p* < 0.001 compared with the STZ + HFD group; *n* = 4 per group.

To examine apoptosis-associated molecular signaling, immunofluorescence staining for the pro-apoptotic protein Bax and the anti-apoptotic protein Bcl-2 was performed (Figure 11). Bax signal was minimal in the C57BL/6 and ApoE^−^/^−^ groups but was markedly intensified in the STZ + HFD group, whereas Bcl-2 immunoreactivity showed the opposite trend, decreasing under glucolipotoxic conditions.

Quantitative analysis confirmed that Bax expression was substantially increased in the STZ + HFD group (13.11-fold of control, *p* < 0.001 vs. ApoE^−^/^−^), with an intermediate elevation observed in the STZ-only group (2.66-fold). Bcl-2 expression was reduced in the STZ + HFD group to 0.50-fold of control. The Bax/Bcl-2 ratio, an established index of mitochondrial apoptotic susceptibility, was markedly elevated in the STZ + HFD group (26.07-fold of control, *p* < 0.001 vs. ApoE^−^/^−^). Melatonin treatment significantly attenuated Bax expression to 6.39-fold (*p* < 0.001 vs. STZ + HFD), restored Bcl-2 expression to 0.89-fold, and lowered the Bax/Bcl-2 ratio to 7.53-fold (*p* < 0.001 vs. STZ + HFD). These findings indicate that melatonin shifts the mitochondrial apoptotic balance toward cardiomyocyte survival under glucolipotoxic conditions.

## 4. Discussion

The present study employed a combined streptozotocin and high-fat diet model in ApoE^−^/^−^ mice to mimic the glucolipotoxic environment of type 2 diabetes with concomitant dyslipidemia. This model reproduces a clinically relevant cardiometabolic context in which hyperglycemia and lipid overload synergistically accelerate myocardial injury. Metabolic disturbances observed in the diabetic groups, including elevated triglycerides, LDL cholesterol, and HbA1c, reflect the combined burden of dyslipidemia and chronic hyperglycemia, both of which contribute to cardiovascular risk in diabetes. Melatonin significantly improved lipid parameters and reduced HbA1c, consistent with evidence that melatonin modulates lipid metabolism and glycemic control through circadian regulation of hepatic metabolic pathways and insulin sensitivity. Clinical meta-analyses have demonstrated reductions in LDL cholesterol and HbA1c following melatonin supplementation, although fasting plasma glucose often remains unchanged [34,35]. The persistence of fasting hyperglycemia despite reduced HbA1c suggests that melatonin preferentially influences postprandial glucose excursions or non-enzymatic glycation processes rather than basal hepatic glucose output. In addition, in the present animal model, body weight remained low in the STZ-containing groups and did not increase despite HFD feeding. This finding is consistent with the diabetogenic effect of STZ, which damages pancreatic β-cells and induces insulin deficiency, thereby impairing energy storage and promoting a catabolic metabolic state [36]. Under severe insulin-deficient conditions, protein turnover is increased and skeletal muscle mass may decline even when caloric intake is adequate [37]. Therefore, the catabolic drive induced by STZ may have outweighed the adipogenic effect of HFD, accounting for the absence of body weight gain in the STZ and STZ + HFD groups.

Notably, heart weight was increased in the STZ and STZ + HFD groups relative to the ApoE^−^/^−^ group. Because these diabetic mice also exhibited reduced body weight, the heart weight/body weight ratio provides a more appropriate index of relative cardiac enlargement. The increased heart weight/body weight ratio in the STZ and STZ + HFD groups suggests pathological cardiac remodeling rather than physiological growth. In diabetic hearts, reactive hypertrophy, interstitial fibrosis, and metabolic stress can contribute to increased cardiac mass during adverse remodeling [38]. Melatonin reduced heart weight and the heart weight/body weight ratio to values comparable to the ApoE^−^/^−^ group, suggesting attenuation of diabetes-associated cardiac remodeling. This structural benefit occurred without normalization of body weight, supporting the possibility that melatonin exerted cardioprotective effects beyond simple changes in body mass.

Electrophysiological impairment is an early manifestation of diabetic cardiac injury/remodeling under glucolipotoxic stress, driven by oxidative modification of ion channels and gap junction proteins. Reduced heart rate, prolonged RR interval, and decreased signal amplitude are consistent with impaired conduction and autonomic dysfunction under diabetic conditions. Oxidative stress-mediated alteration of connexin 43 has been identified as a key mechanism underlying conduction abnormalities in the diabetic myocardium [39]. Melatonin partially restored these electrophysiological parameters, which is consistent with its capacity to preserve mitochondrial membrane potential and reduce reactive oxygen species accumulation, thereby stabilizing cardiomyocyte excitability and electrical conduction [40].

Histopathological alterations further illustrate the impact of glucolipotoxicity on myocardial integrity. Accumulation of glycogen and lipid droplets, together with leukocyte infiltration and myofibrillar disorganization, reflects impaired substrate utilization and lipotoxic stress. Excess fatty acids promote the formation of toxic lipid intermediates such as ceramides and diacylglycerols, which disrupt mitochondrial function and enhance ROS production [41]. These processes activate inflammatory signaling pathways and contribute to structural deterioration. Melatonin markedly reduced inflammatory infiltration and intracellular substrate accumulation, indicating improved metabolic handling and suppression of lipotoxic injury. The inflammatory infiltrate observed on hematoxylin and eosin staining is likely to include macrophage accumulation, a characteristic feature of diabetic cardiomyopathy that contributes to myocardial inflammation and adverse remodeling [42]. The reduction in this infiltrate by melatonin is consistent with attenuated macrophage-associated inflammation in the glucolipotoxic myocardium.

In addition, oxidative DNA damage is a central consequence of sustained reactive oxygen species production in the diabetic heart. Elevated 8-OHdG levels indicate increased genomic instability and oxidative injury [43]. Melatonin significantly reduced 8-OHdG expression, which is consistent with its well-established role as a direct free radical scavenger and an inducer of endogenous antioxidant defenses [44]. In addition to neutralizing reactive oxygen and nitrogen species, melatonin enhances DNA repair capacity through activation of base excision repair enzymes, thereby preserving genomic integrity under oxidative stress conditions. Notably, Nrf2 expression in the glucolipotoxic myocardium remained at levels comparable to those of non-diabetic controls, indicating that the endogenous Nrf2-mediated antioxidant defense failed to mount an adequate response despite escalating oxidative DNA damage. This phenomenon has been described in chronic diabetic conditions, where progressive hyperglycemia and lipotoxicity impair Keap1–Nrf2 signaling and downstream antioxidant gene transcription, leaving the diabetic heart vulnerable to sustained oxidative injury [30]. Melatonin treatment markedly induced Nrf2 expression beyond baseline, consistent with its established role as a pharmacological Nrf2 activator capable of overriding this diabetic suppression. Recent evidence further indicates that melatonin-driven Nrf2 activation suppresses NLRP3 inflammasome assembly under hyperglycemic stress [45], providing a mechanistic link between the antioxidant restoration and the concurrent reduction in inflammasome-related signaling observed in the present study. Combined with melatonin’s reported activation of the SIRT1/PGC-1α/Nrf2 axis [46], these findings support the interpretation that melatonin reinforces the antioxidant frontline through a coordinated transcriptional program rather than merely scavenging free radicals.

Nitrosative stress further amplifies myocardial injury in diabetes. Increased expression of inducible nitric oxide synthase leads to excessive nitric oxide production, which reacts with superoxide to form peroxynitrite, a highly reactive species that damages proteins, lipids, and DNA. This process contributes to mitochondrial dysfunction and sustains inflammatory signaling [47]. Melatonin suppressed iNOS expression, indicating attenuation of nitrosative stress and its downstream pathological effects. This effect is consistent with inhibition of NF-κB-dependent transcriptional activation of iNOS in inflammatory conditions. Diabetic cardiomyopathy is closely associated with chronic low-grade systemic inflammation, in which circulating mediators such as TNF-α, IL-6, CRP, IL-1β, and IL-18 contribute to myocardial inflammation and adverse cardiac remodeling [48].

In addition, inflammatory signaling mediated by the NF-κB/NLRP3 inflammasome axis plays a pivotal role in diabetic cardiac injury/remodeling under glucolipotoxic stress. NF-κB activation primes the expression of NLRP3 and pro-inflammatory cytokines, while mitochondrial reactive oxygen species act as a secondary signal for inflammasome assembly. Activation of caspase-1 subsequently promotes maturation of IL-1β, amplifying sterile inflammation and cardiomyocyte injury [9]. Elevated expression of NF-κB, NLRP3, TNF-α, and IL-1β under glucolipotoxic conditions indicates robust activation of this pathway. Melatonin suppressed all components of this axis, which is consistent with evidence that melatonin inhibits NF-κB signaling through SIRT1-mediated deacetylation and reduces mitochondrial reactive oxygen species, thereby disrupting both priming and activation steps of the inflammasome [49].

Cardiomyocyte apoptosis is a critical driver of progressive myocardial dysfunction. Sustained oxidative stress and inflammatory signaling activate mitochondrial apoptotic pathways, leading to irreversible cell loss. The adult mammalian heart possesses limited regenerative capacity, with cardiomyocyte turnover estimated at less than 1% per year, such that lost cardiomyocytes cannot be effectively replenished after injury [50]. Consequently, even chronic low-level increases in cardiomyocyte apoptosis can accumulate over time and translate into progressive contractile decline, adverse remodeling, and ultimately heart failure [31]. Apoptosis is exceedingly rare in healthy myocardium, but rises markedly under pathological stress, while typically remaining within a sublethal range compatible with chronic disease rather than acute organ-lethal cell loss. The Bax/Bcl-2 ratio functions as a mitochondrial rheostat governing this apoptotic threshold, with elevated ratios driving outer membrane permeabilization, cytochrome c release, and caspase activation [31].

These principles provide a biologically plausible context for the present findings. In the STZ + HFD group, the Bax/Bcl-2 ratio was elevated approximately 26-fold relative to control, accompanied by a corresponding increase in TUNEL-positive cardiomyocytes. Despite this pronounced shift in the apoptotic set-point, animals remained viable throughout the 8-week experimental period, indicating that the apoptotic burden was sufficient to drive measurable structural and functional impairment but did not reach the threshold of fulminant cardiomyocyte loss that would preclude short-term survival. This pattern is consistent with the chronic and progressive nature of diabetic cardiomyopathy in patients, in whom cumulative cardiomyocyte attrition develops over years rather than abruptly [31]. Melatonin treatment significantly reduced both TUNEL positivity and the Bax/Bcl-2 ratio (lowered to approximately 7.5-fold of control), indicating partial restoration of the mitochondrial apoptotic set-point toward a more survival-compatible state. By attenuating this cumulative apoptotic burden, melatonin is expected to preserve functional cardiomyocyte mass over the long-term course of glucolipotoxic injury, consistent with previous findings demonstrating its ability to modulate mitochondrial integrity, suppress caspase activation, and regulate apoptosis-related signaling pathways [32,46].

ANP and BNP are structurally related cardiac natriuretic peptides that signal through their shared receptor, natriuretic peptide receptor-A (NPR-A) and exert overlapping counter-regulatory roles in the stressed heart, including vasodilation, natriuresis, inhibition of the renin–angiotensin–aldosterone system, and suppression of pathological hypertrophy and fibrosis [51]. Despite shared receptor coupling, they differ in their predominant sites of synthesis and primary secretory stimuli: ANP is produced predominantly by atrial cardiomyocytes in response to atrial wall stretch from volume overload, whereas BNP is synthesized principally in ventricular cardiomyocytes under pathological conditions in response to increased wall stress from either pressure or volume overload [52]. Both peptides are therefore regarded as complementary indices of myocardial strain in DCM, with elevated circulating levels reflecting the magnitude of structural and hemodynamic derangement [29]. Consistent with this framework, both ANP and BNP were significantly upregulated in glucolipotoxicity groups relative to controls, with peak expression observed under combined glucolipotoxicity conditions, suggesting that concurrent dyslipidemia compounds the hemodynamic burden imposed by hyperglycemia alone. Sustained natriuretic peptide overexpression in the diabetic myocardium reflects a compensatory but insufficient counter-regulatory response to maladaptive remodeling, indicating that endogenous NPR-A signaling is unable to arrest the structural deterioration under chronic glucolipotoxic stress [53]. Melatonin treatment significantly attenuated both ANP and BNP expression in the melatonin treatment group, and this reduction occurred without normalization of blood glucose, supporting the interpretation that melatonin suppresses the natriuretic stress burden through attenuation of upstream oxidative and inflammatory signaling independently of glycemic control. The parallel reduction in ANP and BNP further corroborates the histological evidence of attenuated cardiomyocyte hypertrophy and fibrotic remodeling [54].

Several limitations should be acknowledged. First, the relatively small sample size may limit the statistical power and generalizability of the findings. In addition, tibia length was not measured; therefore, the heart weight/tibia length ratio could not be calculated. Future studies should include HW/TL analysis, cardiomyocyte cross-sectional area, and fibrosis quantification to more directly evaluate cardiac hypertrophy and structural remodeling. Second, cardiac function was not directly assessed by echocardiography or hemodynamic measurements; therefore, the present results should be interpreted as evidence of melatonin-mediated attenuation of diabetic cardiac injury and remodeling rather than definitive functional recovery of diabetic cardiomyopathy. In addition, although ECG analysis demonstrated glucolipotoxicity-induced alterations in cardiac electrical conduction and partial improvement after melatonin treatment, echocardiographic assessment was not performed in the present study. Therefore, whether these electrical abnormalities are accompanied by impaired systolic or diastolic cardiac function remains to be determined. Future studies incorporating echocardiography are required to directly evaluate the functional consequences of these ECG changes. Third, because ApoE^−^/^−^ mice exhibit severe dyslipidemia and atherosclerotic burden, potential vascular or coronary contributions to cardiac injury cannot be excluded. Finally, this study used only male mice and did not include a melatonin-only group, dose-response design, or circadian timing assessment; future studies addressing these issues, together with functional cardiac evaluation and direct molecular validation, are warranted to strengthen the translational relevance of melatonin in diabetic cardiac injury. Circulating inflammatory cytokines such as TNF-α, IL-6, CRP, IL-1β, and IL-18 were not directly measured, and the inflammatory evidence presented here is based on cardiac tissue expression; serum or plasma cytokine profiling would help link systemic inflammation to the cardiac phenotype. Macrophage-specific immunostaining, such as F4/80 or CD68, was also not performed; future work should include F4/80 or CD68 immunohistochemistry to quantify cardiac macrophage infiltration and to clarify whether melatonin modulates macrophage recruitment, polarization, or resolution in the diabetic heart. In addition, the murine dose employed in this study was 20 mg/kg/day, administered orally for 8 weeks. Translation to human application requires allometric scaling based on body surface area, as recommended by current regulatory guidelines [55]. Applying the Km-factor method (HED = animal dose × Km~mouse~/Km~human~ = 20 × 3/37), the estimated human equivalent dose (HED) is approximately 1.62 mg/kg/day, corresponding to roughly 97 mg/day for a 60-kg adult. This value exceeds the dose range conventionally employed in clinical investigations, where melatonin is typically administered at 3–20 mg/day [56]. This numerical discrepancy, however, does not preclude translational relevance. Melatonin possesses pharmacokinetic and pharmacodynamic properties that weaken the assumption underlying linear interspecies dose transposition. It preferentially accumulates within mitochondria via oligopeptide transporter-mediated uptake, achieving intraorganellar concentrations substantially exceeding those in plasma [57]. At this subcellular site, melatonin acts as a direct radical scavenger through receptor-independent mechanisms, exerting antioxidant actions at the primary locus of ROS generation [56]. These characteristics, together with its pleiotropic modulation of NF-κB and NLRP3 inflammasome signaling, enable biological efficacy at tissue concentrations lower than those predicted by body surface area normalization alone. Accordingly, the calculated HED should be interpreted as a theoretical reference rather than a direct clinical dose recommendation. Future dose-finding studies are required to define the optimal therapeutic range of melatonin for diabetic cardiac injury/remodeling under glucolipotoxic stress, with attention to long-term safety and efficacy in human subjects. Although IHC and IF analyses allowed for visualization of the spatial distribution and relative expression of inflammatory, pyroptosis-associated, and apoptosis-associated markers in cardiac tissue, these approaches do not provide absolute quantification of protein abundance. Western blotting or other biochemical assays would strengthen the mechanistic interpretation. Therefore, the present findings should be interpreted as histology-based evidence supporting an association between melatonin treatment and attenuation of oxidative stress-, inflammation-, pyroptosis-, and apoptosis-associated myocardial injury. Future studies incorporating Western blotting, enzyme activity assays, or loss-of-function approaches will be required to further validate the proposed mechanistic pathway. In addition, previous studies have reported the cardiometabolic benefits of melatonin in diabetic models, although the present study did not include an STZ + MT group. Abdulwahab et al. demonstrated that melatonin protected the diabetic heart against oxidative stress, inflammation, and apoptosis in a rat model of diabetes [58]. Alsharif et al. further showed that melatonin attenuated streptozotocin-induced metabolic disturbances and improved the systemic redox balance in diabetic rats [59]. These findings support the cardiometabolic protective potential of melatonin under diabetic conditions. The absence of an STZ + MT group precludes a direct comparison of melatonin under hyperglycemia alone with that under combined hyperglycemic and dyslipidemic stress. Future studies incorporating an STZ + MT group are warranted to distinguish the cardioprotective effects of melatonin between these metabolic conditions.

## 5. Conclusions

Melatonin attenuates myocardial injury under glucolipotoxic conditions through coordinated suppression of oxidative stress, inhibition of NF-κB/NLRP3 inflammasome activation, reduction in cardiomyocyte apoptosis, and attenuation of fibrotic remodeling. These protective effects occur independently of fasting glucose normalization, indicating a glucose-independent mechanism targeting downstream pathological pathways. These findings support melatonin as a multi-target therapeutic candidate for adjunctive intervention in diabetic cardiac injury/remodeling under glucolipotoxic stress.

## Figures and Tables

**Table 1:** The effect of melatonin on the blood analysis in the STZ-diabetic ApoE^−^/^−^ mice.

	C57BL/6	ApoE^−^/^−^	STZ	STZ + HFD	STZ + HFD + MT
CHO (mg/dL)	94.6 ± 17.32 ***	822.6 ± 100.25	1420.8 ± 68.07 ***	1518.8 ± 102.26 ***	1435.6 ± 36.04 ***
TG (mg/dL)	71.0 ± 11.46	86.8 ± 14.60	84.6 ± 13.25	136.2 ± 16.60 **	89.6 ± 12.81 ^##^
HDL (mg/dL)	57.8 ± 6.87 ***	104.6 ± 14.29	105.0 ± 9.30	122.4 ± 25.65	112.2 ± 12.15
LDL (mg/dL)	10.8 ± 2.28 ***	111.8 ± 9.36	132.6 ± 18.31	244.6 ± 43.46 ***	157.6 ± 42.12 ^##^
HbA1c (%)	4.0 ± 0.18	3.9 ± 0.08	5.4 ± 1.68	6.8 ± 0.70 ***	5.1 ± 0.76 ^##^

C57BL/6 mice were fed a normal diet as the control group. ApoE^−^/^−^ mice were fed a normal diet as the negative control group. STZ-diabetic ApoE^−^/^−^ mice were induced by intraperitoneal injection of streptozotocin. The STZ + HFD group received STZ injection combined with a high-fat diet, and the STZ + HFD + MT group received STZ injection, high-fat diet, and oral administration of melatonin at 20 mg/kg for 8 weeks. Blood biochemical parameters, including total cholesterol (CHO), triglyceride (TG), high-density lipoprotein cholesterol (HDL), low-density lipoprotein cholesterol (LDL), and HbA1c, were measured. Data are expressed as mean ± SEM. Statistical analysis was performed using SigmaPlot 12.0. ** *p* < 0.01 and *** *p* < 0.001 compared with the ApoE^−^/^−^ group; ^##^ *p* < 0.01 compared with the STZ + HFD group; *n* = 4 per group.

**Table 2:** Melatonin ameliorated the ECG parameters in the STZ-diabetic ApoE^−^/^−^ mice.

	C57BL/6	ApoE^−^/^−^	STZ	STZ + HFD	STZ + HFD + MT
Heart Rate (bpm)	551.6 ± 1.14 **	562 ± 2.55	513 ± 1.58 ***	451.8 ± 0.84 ***	524.8 ± 0.84 ^###^
RR interval (ms)	107.33 ± 1.49	106 ± 0.91	115.33 ± 0.75 ***	130.67 ± 0.91 ***	112 ± 0.75 ^###^
PR interval (ms)	34.67 ± 2.98	30.33 ± 3.61	43 ± 0.75	31.7 ± 2.65	41.33 ± 3.21 ^###^
QRS interval (ms)	16.33 ± 2.47	19 ± 2.53	16.67 ± 1.18	15.91 ± 2.34	19 ± 3.25
P amplitude (mV)	0.1 ± 0.02 **	0.14 ± 0.02	0.1 ± 0.01 **	0.07 ± 0.01 ***	0.1 ± 0.02 ^#^
R amplitude (mV)	0.65 ± 0.05 ***	0.43 ± 0.02	0.32 ± 0.01 ***	0.5 ± 0.03 ***	0.59 ± 0.04 ^###^

Electrocardiographic parameters, including heart rate, RR interval, PR interval, QRS interval, P amplitude, and R amplitude, were measured in C57BL/6, ApoE^−^/^−^, STZ, STZ + HFD, and STZ + HFD + MT groups. STZ + HFD-induced diabetic ApoE^−^/^−^ mice exhibited altered ECG parameters, including decreased heart rate and increased RR interval, whereas melatonin treatment improved ECG abnormalities. Data are expressed as mean ± SEM. Statistical analysis was performed by ANOVA followed by Duncan’s multiple range test. ** *p* < 0.01 and *** *p* < 0.001 compared with the ApoE^−^/^−^ group; ^#^ *p* < 0.05 and ^###^ *p* < 0.001 compared with the STZ + HFD group.

## Data Availability

The data used to support the findings of the study are available upon reasonable request from the corresponding author.

## Supplementary Materials

The following supporting information can be downloaded at https://www.mdpi.com/article/10.3390/antiox15070825/s1. Table S1: Primary antibodies; Table S2: Chemicals, reagents and kits.

## Abbreviations

The following abbreviations are used in this manuscript:

8-OHdG	8-hydroxy-2′-deoxyguanosine
ANP	Atrial natriuretic peptide
ApoE	Apolipoprotein E
ApoE^−^/^−^	Apolipoprotein E-deficient
BNP	Brain natriuretic peptide
CHO	Total cholesterol
DAB	Diaminobenzidine
DAPI	4′,6-diamidino-2-phenylindole
DCM	Diabetic cardiomyopathy
DM	Diabetes mellitus
ECG	Electrocardiography
FBS	Fetal bovine serum
GSDMD	Gasdermin D
H&E	Hematoxylin and eosin
HbA1c	Glycated hemoglobin
HDL	High-density lipoprotein
HDL-C	High-density lipoprotein cholesterol
HFD	High-fat diet
IL-1β	Interleukin-1β
iNOS	Inducible nitric oxide synthase
IOD	Integrated optical density
LDL	Low-density lipoprotein
LDL-C	Low-density lipoprotein cholesterol
MT	Melatonin
NF-κB	Nuclear factor-κB
NLRP3	NLR family pyrin domain containing 3
Nrf2	Nuclear factor erythroid 2-related factor 2
PAS	Periodic acid–Schiff
PBS	Phosphate-buffered saline
ROS	Reactive oxygen species
SD	Standard deviation
STZ	Streptozotocin
STZ + HFD	Streptozotocin plus high-fat diet
STZ + HFD + MT	Streptozotocin plus high-fat diet plus melatonin
TBST	Tris-buffered saline with Tween 20
TdT	Terminal deoxynucleotidyl transferase
TG	Triglyceride
TNF-α	Tumor necrosis factor-α
TUNEL	Terminal deoxynucleotidyl transferase dUTP nick-end labeling
