# Supplementary material for: Melatonin Attenuates Glucolipotoxicity-Induced Cardiac Oxidative Stress, Inflammation, Pyroptosis, and Fibrotic Remodeling in STZ/HFD-Treated ApoE−/− Mice
Source: Antioxidants (Basel). 2026 Jun 30;15(7):825. doi: 10.3390/antiox15070825 (PMC13404163; doi:10.3390/antiox15070825)
Supplement: Supplementary file 1 [file antioxidants-15-00825-s001.zip › antioxidants-4342688-supplementary.pdf]

Table S1. Primary antibodies

Target	Source / vendor	Catalog no.	Dilution	RRID
8-OHdG	Abcam	ab48508	1:200	RRID:AB_867461
Nrf2	Santa Cruz Biotechnology	sc-722	1:100	RRID:AB_2108502
NF-κB	Santa Cruz Biotechnology	sc-8008	1:100	RRID:AB_628017
NLRP3	Cell Signaling Technology	15101 (clone D4D8T)	1:200	RRID:AB_2722591
Caspase-1	ABclonal	A23429	1:100	RRID:AB_3683536
GSDMD-N	Cell Signaling Technology	36425	1:200	RRID:AB_2799099
IL-1β	Abcam	ab283818	1:200	RRID:AB_3076536
TNF-α	Novus Biologicals	NBP1-19532	1:200	RRID:AB_1643202
Bax	Santa Cruz Biotechnology	sc-526	1:100	RRID:AB_2064668
Bcl-2	Santa Cruz Biotechnology	sc-492	1:100	RRID:AB_2064290
ANP	Abcam	ab225844	1:200	not found; verify in Antibody Registry
BNP	Abcam	ab239510	1:200	not found; product discontinued; verify
HO-1	Abcam	ab68477	1:200	RRID:AB_11156457
iNOS	Abcam	ab178945	1:200	RRID:AB_2861417
β-actin (loading)	Novus / Sigma-Aldrich	NB600-501H / A5441	1:5000	NB600-501H: RRID:AB_1216153; A5441: RRID:AB_476744
α-tubulin (loading)	Elabscience	E-AB20069	1:5000	RRID:AB_3750902

Table S2. Chemicals, reagents and kits

Reagent / kit	Vendor	Catalog no. / CAS
Streptozotocin (STZ)	Sigma-Aldrich	S0130
Melatonin	Sigma-Aldrich	M5250
Sodium citrate buffer (STZ vehicle)	Sigma-Aldrich	Sodium citrate tribasic dihydrate S4641 / citric acid monohydrate C1909
Dimethyl sulfoxide (DMSO)	Sigma-Aldrich	D2050
Paraformaldehyde (PFA)	Sigma-Aldrich	P6148
Phosphate-buffered saline (DPBS)	Gibco	21600-010
Triton X-100	Sigma-Aldrich	T8787
Tween-20	Sigma-Aldrich	P1379
Acrylamide / bis-acrylamide	MD Bio	11049
Sodium dodecyl sulfate (SDS)	Amresco	CAS 151-21-3
Tris base	Uni Region Biotech	CAS 77-86-1
TEMED	Alfa Aesar	L00847
Ammonium persulfate (APS)	Sigma-Aldrich	A3678
Glycine	Fisher Scientific	G46-500
PVDF membrane	Millipore	IPVH00010
Nitrocellulose membrane	Pall	T02408
Prestained protein ladder	Thermo Fisher	26616
ECL luminol reagent	Millipore	WBKLS0500
Protein assay kit (BCA / Bradford)	Thermo Fisher Scientific	Pierce BCA Protein Assay Kit, 23227
In Situ Cell Death Detection Kit (TUNEL)	Roche	11684817910
Total cholesterol assay kit	Abcam	ab65390
HDL and LDL/VLDL cholesterol assay kit	Abcam	ab65390
Triglyceride assay kit	Abcam	ab65336
Mouse HbA1c assay kit	Crystal Chem	80310
Ultra Sensitive Mouse Insulin ELISA Kit	Crystal Chem	90080
Hematoxylin and eosin staining reagents	Sigma-Aldrich	Hematoxylin H3136 / Eosin Y E4009
Masson’s trichrome staining kit	Sigma-Aldrich	HT15
Periodic acid–Schiff staining kit	Sigma-Aldrich	395B
DAB substrate kit	Vector Laboratories	SK-4100
Antigen retrieval citrate buffer	Vector Laboratories	H-3300 or equivalent
Mounting medium with DAPI	Vector Laboratories	H-1200
